# Determinants of intermittent preventive treatment with sulfadoxine–pyrimethamine in pregnant women (IPTp-SP) in Mali, a household survey

**DOI:** 10.1186/s12936-021-03764-5

**Published:** 2021-05-22

**Authors:** Oumar Sangho, Moctar Tounkara, Lillian Joyce Whiting-Collins, Madeleine Beebe, Peter J. Winch, Seydou Doumbia

**Affiliations:** 1 Department of Education and Research of Biological and Medical Sciences, Faculty of Pharmacy, University of Sciences, Techniques and Technologies of Bamako, Bamako, Mali; 2 Department of Education and Research in Public Health and Specialties, Faculty of Medicine and Dentistry, University of Sciences, Techniques and Technologies of Bamako, Bamako, Mali; 3 Department of International Health, Johns Hopkins Bloomberg School of Public Health, Baltimore, MD USA

**Keywords:** Access to care, Geographic access, Intermittent preventive treatment, Malaria in pregnancy, Antenatal care

## Abstract

**Background:**

In malaria endemic regions, intermittent preventive treatment with sulfadoxine-pyrimethamine (IPTp-SP) is recommended for all pregnant women during prenatal consultation, from the fourth month of pregnancy up to the time of delivery. The Government of Mali is aiming for universal coverage of IPTp-SP. However, coverage is still low, estimated to be 18% for completion of three doses (IPTp-SP3). The objective of this study was to identify the factors that influence IPTp-SP adherence in the Health District of Sélingué, Mali.

**Methods:**

This was a cross-sectional survey with 30 clusters, proportional to village size, with two stages of sampling. Data were collected electronically with Magpi software during face-to-face interviews/surveys. The data were analysed with SPSS version 20. A descriptive analysis and bivariate and multivariate logistic regression were performed. An equity analysis examined the effect of distance from health care facility on completion of three or more antenatal visits (ANC3 +) and three or more doses of intermittent preventive treatment (IPTp-SP3 +).

**Results:**

Of the 1,021 women surveyed, 87.8% (n = 896) attended at least one ANC visit. Of these, 86.3% (n = 773) received at least one dose of IPTp-SP. Compliance with three or more doses of IPTp-SP was 63.7%. The determinants statistically related to ANC3 + were the early initiation of ANC (OR = 3.22 [1.22, 10.78]), and the presence of a community health centre (CHC) in the village (OR = 9.69 [1.09, 86.21]). The ability to read (OR = 1.60 [1.01, 2.55]), the early initiation of ANC (OR = 1.46 [1.06, 2.00], knowledge of the utility of the drug (OR = 2.38 [1.24, 4.57]), and knowledge of the recommended dose of the drug (OR = 6.11 [3.98, 9.39]) were related to completion of three or more treatments (IPTp-SP3 +).

**Conclusion:**

The early initiation of ANC was a positive determinant of the completeness of both ANC3 + and IPTp-SP3 + . This study shows that a successful implementation of the IPTp strategy can be achieved by improving access to prenatal care at community health facilities, and strengthening patient-provider communication to ensure adequate knowledge on dosing of IPTp-SP and the benefits to mother and child.

## Background

Malaria is a major public health problem. In 2019, the number of malaria cases was estimated at 229 million [1]. Of these cases, 94% were recorded by the World Health Organization (WHO) Africa region, followed by the South-East Asia region (3%) [1]. Of the 409,000 malaria deaths worldwide, 51% were recorded in seven African countries [1]. Malaria is especially detrimental to the health and well-being of women and children [2]. It can lead to maternal anaemia, which increases the risk of haemorrhage during childbirth [2]. In some endemic areas, malaria can directly contribute up to 25% of all maternal death [2]. Malaria during pregnancy also increases the risk of miscarriage and affects foetal development, as well as preterm birth and low birth weight [3]. In 2019, 33 million pregnancies were estimated in malaria-endemic areas in Africa, of which 35% (12 million) were exposed to malaria infection [1].

As part of antenatal care (ANC) services, every effort should be made to improve access to sulfadoxine-pyrimethamine intermittent preventive treatment (IPTp-SP) everywhere in Africa where malaria endemicity level ranges from moderate to high [4–6]. The WHO recommends at least four ANC visits during pregnancy [4, 6]. Starting as early as possible in the second trimester, IPTp-SP is recommended for all pregnant women at each ANC visit until delivery, and doses should be administered at least one month apart [6]. Sulfadoxine-pyrimethamine (SP) should not be administered during the first trimester of pregnancy [6]. The last dose of IPTp-SP can be administered up to the time of delivery without any safety concern [4, 6]. IPTp-SP should preferably be administered in the form of three SP tablets, under direct observation, which is the total required dosage of 1500 mg/75 mg, and can be administered with or without food [6]. SP should not be given to women receiving prophylactic treatment with cotrimoxazole because of a higher risk of adverse events [6].

Globally, the percentage of women receiving IPTp-SP during pregnancy has increased over the years, although it remains below targets of 80% as cited in Yaya et al*.* [7] and below the target of universal coverage of all pregnant women attended ANC services [8]. IPTp-SP has been adopted by 37 countries [8]. In 2019, 80% of pregnant women used ANC services at least once. The percentage of pregnant women who received IPTp-SP1 + , IPTp-SP2 + and IPTp-SP3 + were 62%, 49% and 34% respectively [1]. A study using data from the Malaria Indicator Surveys (MIS) conducted in eight Sub-Saharan African countries found the overall prevalence of 29.5% (95% CI = 28.2–30.5) for IPTp-SP3 + in 2018 [7].

In Mali, the objective of the National Policy for Malaria Control, led by the national malaria control programme ('Programme National de Lutte contre le Paludisme', PNLP), is to achieve universal coverage of SP through free distribution to pregnant women countrywide [9]. The main goal of the 2013–2017 Strategic Plan of the PNLP [5] was to ensure universal access to prevention measures for 100% of the population at risk of malaria, including the use of IPT by pregnant women [5].

In the Malian healthcare system, ANC provides women with the opportunity to get IPTp-SP at no cost. In 2014, 74% of Malian pregnant women attended ANC1 while 41% attended ANC4 and above (ANC4 +) [10]. In the Sikasso region, there was 79% coverage for ANC1 [10]. In Sélingué, in 2017, the frequencies of ANC1, ANC3 and ANC4 were 89%, 26% and 28%, respectively [11].

Hurley's secondary analysis of the 2012–2013 Demographic and Health Survey (DHSM-V) data [10] shows that 56.2% and 29.9% of recently pregnant women benefited from IPTp-SP1 and TPIg- SP2 + , respectively, in Mali [12]. The MIS conducted in Mali in 2015 shows adherence of 66.4% for IPTp-SP1, 44.4% for IPTp-SP2 and 21% for IPTp-SP3 + [13].

In Mali’s Sikasso Region, the DHS found adherence of IPTp-SP1 to be 68.42% [14]. Adherence IPTp-SP2 and IPTp-SP3 + were 50.8% and 26.8%, respectively [14]. In 2015, the MIS showed an adherence of 68.7%, 44.4% and 22.2%, respectively for IPTp-SP1, IPTp-SP2 and IPTp-SP3 + [13]. In Sélingué, in 2016, 72% of pregnant women had received IPTp-SP1 compared to 43% for IPTp-SP2 and only 19% for IPTp-SP3 [11].

Based on these data, the goals of the PNLP have not been achieved.

Several studies have explored the determinants of the use and completeness of IPTp-SP [12, 15–31]. Non-attendance and/or completeness of ANC is considered a key determinant of IPTp-SP coverage [11, 15, 20, 24]. A qualitative study conducted in Mali in 2013 identified the following factors influencing the completeness of two doses of IPTp-SP: late use or non-use of ANC services, perception of malaria during pregnancy, poor acceptability of SP, stock-outs of SP, and insufficient information on the policy of providing SP free of cost to pregnant women [15]. In spite of this free IPTp national distribution policy, the authors reported variations in IPTp costs at various health facilities and during prenatal visits [15]. Lack of education and economic wellbeing have also been identified as factors influencing the use of IPTp-SP, with high level of education, high level of economic well-being, and living in an urban area strongly associated with women taking IPTp-SP [23]. In 2013, a meta-analysis was done by Hill et al*.* [24]. The results suggested that barriers to the use of IPTp-SP included lack of clarification of guidelines and policy, poor organization of services, stock-outs, costs of health services, lack of competent health workers, and the underuse of ANC by women [24]. Key determinants of IPTp-SP were level of education, knowledge of malaria and IPTp-SP, socio-economic status, parity, and number of and early initiation of ANC visits [24]. Despite these factors affecting completeness of IPTp-SP, frequencies remain low. It is necessary to find an alternative strategy to improve IPTp-SP coverage. Sélingué is a malaria endemic, irrigated rice-growing area with a malaria incidence of 16.9% [11]. This study was conducted to identify the factors that specifically influence IPTp-SP in the Sélingué Health District in an effort to inform local efforts to improve IPTp-SP.

## Methods

### Site

The study was conducted in 2016, from January 11^th^ to February 9^th^ in the health district of Sélingué, located 120 km southwest of Bamako. The health district of Sélingué is composed of seven subdistricts: Kangaré, Binko, Siékorolé, Tanga, Carrière, Diarani and Faraba and has three private clinics. The population in 2012 was 91,425 across 60 villages [32]. The dam on the Sankarani River, a branch of the Niger River, has created an artificial lake of 409 km^2^ in Sélingué, making rice cultivation, gardening, and fishing the main activities in the area [33]. In 2016, there were 18 community health workers (CHW) in the district, each working with several community relays (CR) and traditional birth attendants in each village [34]. CHWs were involved in minor medical care for children under five years of age and in the education and referral of pregnant women to ANC services.

### Design

This was a cross-sectional survey with cluster sampling proportional to village population size at two levels. Thus, the number of clusters per village depended on the population size in each village. A total of 30 clusters were needed to have a representative sample. Overall, 960 households were selected for the 30 clusters (each cluster consisted of 32 households with at least one eligible woman). The minimum sample size was 981 households. To be eligible, the woman must have had a pregnancy in the two years prior to the survey, regardless of pregnancy outcome, according to the same selection process used in the MIS and the sixth Demographic and Health Surveys (DHS-VI) [13, 14]. Participants were interviewed face-to-face by interviewers and were shown the three SP tablets to see if they recognized them.

### Data quality management and control

All data were collected on tablets by six data collectors and two supervisors (enumerators) over one month, using Magpi remote data collection software [35]. Data collectors were trained by the supervisors. The tools were pre-tested in Diago, a village near Bamako. Data were checked by the enumerators, uploaded to a secure cloud-based server, and later exported to an Excel spreadsheet by the data manager. Data were then analysed in SPSS software version 20 [36].

### Data analysis

Analysis focused on the following two outcomes:

#### Completion of three or more antenatal consultations (ANC3 +)

**Fig. 1:**
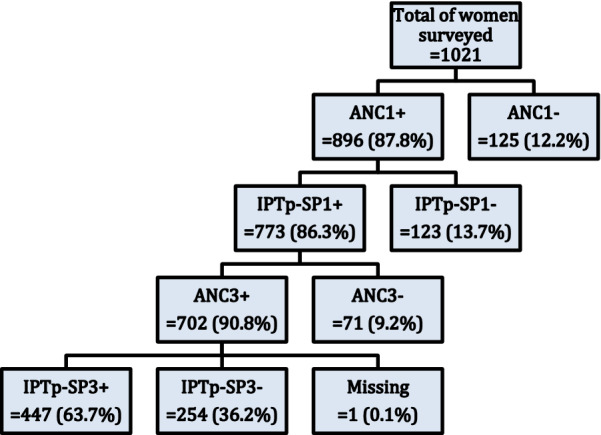
Flow diagram of IPTp-SP intake among women surveyed in 2016, Sélingué, Mali

Women who completed at least three ANC visits were coded 1, and those who did not were coded 0. These women should have received IPTp-SP1 + (Fig. 1). Only women who achieved ANC3 + were considered, because with the current national policy, a woman may have obtained all three doses of IPTp-SP without completing the ANC4 + .

#### IPTp-SP3 + intake

Women who received at least three doses of IPTp-SP were coded 1, and 0 if they did not receive the three doses. These women were those who have completed their ANC3 + (Fig. 1).

Bivariate analyses were conducted according to the flow presented in Fig. 1. Bivariate analysis for the outcome of IPTp-SP3 + intake included women who completed ANC1, IPTp-SP1 and ANC3 + and was performed using Chi square tests and simple logistic regression. Odds ratios (OR) were estimated with 95% confidence intervals (95% CI) and the association between dependent and independent variables (age, level of education, parity, distance from the village to CHC, literacy, age of pregnancy at the first ANC, knowledge of the usefulness of SP, knowledge of the recommended SP dose, knowledge of the period of starting taking SP, number of SP payments, given ITN in ANC, presence of CHC in the village, rurality of the village of residence, poverty quintile) was verified using the Kendall tau B correlation [37]. Variables that had a significant association during the bivariate analysis were selected for the multivariate (global) model. Multiple logistic regression was performed and adjusted odds ratios (ORa) were presented with a p value of 0.05. Correlation between the independent variables with Kendall's tau B was assessed and variables that demonstrated significant correlations were eliminated. For ANC3 + , the variable "ability to read", due to its importance for understanding of medical information, was added.

To measure equity in terms of geographic accessibility to facilities providing ANC, the Gini index and the concentration curve (CC) were used. These data analysis tools are recognized as standard measures employed by health economists to estimate wealth inequity on various health indicators [38–45]. These tools were adapted in this study to estimate inequities in health services utilization according to geographic accessibility. Geographic accessibility was measured using the distance index between a woman’s village of residence and the CHC. This index places women's villages of residence individually on a continuous scale of relative distance. Villages have been grouped into five distance quintiles. Distance quintiles were used to compare the influence of distance on ANC3 + and IPTp-SP3 + . Principal component and factor analysis were used to determine quintiles of poverty. The original line (oblique or diagonal) shows perfect equity. The more the second line curves away from the perfect equity line, the higher the degree of inequity. A curve below the equity line indicates a disproportionate use of services for households from villages close to health facilities. Although the concentration curve is a useful tool for the graphical representation of inequity, it does not quantify the magnitude of inequity. Hence the use of The Gini Index, which is a quantitative measure of inequity in the use of health care. It was used by Wagstaff & van Doorslaer in 2004 to measure the degree of inequity associated with household wealth [45]. The value of the index varies between − 1 and + 1. A value of 0 indicates that the use of health services is equitably distributed among socio-economic groups [46]. In this case, the confidence interval around the index value includes zero. If zero is not within the confidence interval, there is a statistically significant inequity in the use of health services [46]. A negative value of the concentration index implies greater use among the more remote health facilities while a positive value indicates that women in villages around health facilities have greater coverage than women far from health facilities.

## Results

The analysis included 1021 women. Table 1 shows the socio-demographic characteristics of the study sample. More than a quarter of women surveyed (26.2%) were between 20–24 years old. Many women (68.7%) had no level of education. Multiparas were the most frequent with 61.1%. Most of the women surveyed (73.4%) lived within 5 km of a CHC. Figure 1 shows the flow chart of the completeness of ANC3 + and IPTp-SP3 + . Among the 447 women who completed IPTp-SP3 + , about half (223) took SP under Direct Observed Therapy (DOT). One hundred of those women said they brought their own water to the centre for the DOT. Only 96 of the 223 affirmed they took SP on empty stomach. Among the other half (224) who carried their SP home, 215 (96%) said they took it immediately when they got home.

**Table 1:** Sociodemographic characteristics of women surveyed in 2016 Sélingué District, Mali (N = 1021)

Sociodemographic Variables	n	%
Age groups		
15–19	247	24.2
20–24	268	26.2
25–29	212	20.8
30–34	154	15.1
35–39	103	10.1
40–49	37	3.6
Level of education		
No level of education	701	68.7
Primary 1	213	20.9
Primary 2	88	8.6
At least secondary level	19	1.9
Parity		
Primipara	199	19.5
Secondi parous	197	19.3
Multiparous	625	61.2
Geographic accessibility		
Distance from the village to CHC		
0 to 5 km	749	73.4
6 to 15 km	169	16.6
> 15 km	103	10.1

Sixty five percent of women who completed IPTp-SP3 + mentioned that they were asked if they had eaten before coming to the centre, and 78.1% affirmed the availability of water for the DOT.

### Bivariate and multivariate analysis

Tables 2 and 3 present results from the bivariate and multivariate analyses for ANC3 + and IPTp-SP3 + . Kendall tau B analysis revealed a negative correlation (− 0.048) between the presence of CHC in the woman's village of residence and the rurality of the village of residence, p = 0.175. There was a positive correlation (0.131) between knowledge of the recommended SP dose and knowledge of when to start taking SP, p < 0.0001. There was a positive correlation (0.432) between knowledge of the recommended SP dose and the number of payments for SP, p < 0.0001. Knowledge of the recommended dose was correlated to two variables. Therefore, it was removed from the multivariate analysis presented in Table 2. Similar analysis for IPTp-SP3 + was performed. There was a positive correlation (0.121) between knowledge of the benefit of taking SP and the knowledge of the recommended dose, p = 0.001. There was a positive correlation (0.441) between knowledge of the recommended doses of SP and the number of payments, p < 0.0001. Therefore, knowledge of the recommended dose was removed from the multivariate analysis for IPTp-SP3 + from Table 3.

**Table 2:** Bivariate and multivariate analysis: Predictive variables of ANC3 + among women surveyed in 2016, Sélingué, Mali

Characteristics	n	ANC3 + n (%)	OR [CI 95%]	ORa [CI 95%]
Can read	773			
No	661	599 (90.6)	-	1
Yes	112	103 (92.0)	-	1.04 [0.2; 5.56]
Age of pregnancy at the first ANC	757			
4 months or more	444	385 (86.7)	1	1
3 months or less	313	306 (97.8)	6.7*** [3.02; 14.88]	3.62* [1.22; 10.78]
Knowledge of the recommended SP dose	773			
Incorrect answer	538	476 (88.5)	1	-
Know the recommended dose	235	226 (96.2)	3.27*** [1.6; 6.7]	-
Knowledge of the period of starting taking SP	773			
Incorrect answer	665	597 (89.8)	1	1
4 months or less	108	105 (97.2)	3.99* [1.23; 12.91]	5.41 [0.67; 43.93]
SP payment number	280			
1 time	112	84 (75.0)	1	1
2 times	71	70 (98.6)	23.33** [3.1; 175.84]	21,5** [2.64; 175.09]
3 times or more	97	95 (97.9)	15.83*** [3.66; 68.47]	11,24** [2.5; 50.46]
Given ITN in ANC	772			
No	210	183 (87.1)	1	1
Yes	562	519 (92.3)	1.78* [1.07; 2.97]	1,51 [0.62; 3.7]
Presence of CHC in the village	773			
CHC planned but not operational	22	17 (77.3)	1	1
Village with CHC	282	257 (91.1)	3.02* [1.03; 8.89]	9,69* [1.09; 86.21]
No planned or operational CHC	469	428 (91.3)	3.07* [1.08; 8.75]	7,8 [0.98; 62.4]
Rurality of the village of residence	773			
Rural	448	420 (93.8)	1	1
Less rural	325	282 (86.8)	0.44** [0.27; 0.72]	0.69 [0.27; 1.77]

^*^ = p < 0,05; ** = p < 0,01; *** = p ≤ 0,001

OR = Odds Ratio. ORa = Adjusted Odds Ratio

**Table 3:** Bivariate and multivariate analysis: Predictive variables of IPTp-SP3 + among women surveyed in 2016, Sélingué, Mali

Characteristics	n	IPTp-SP3 + n (%)	OR [CI 95%]	ORa [CI 95%]
Poverty quintile	697			
Q1 (poorest)	128	85 (66.4)	1	1
Q2 (poorer)	140	92 (65.7)	0.97 [0.58; 1.61]	1.5 [0.24; 9.25]
Q3 (mean)	142	100 (70.4)	1.20 [0.72; 2.01]	1.23 [0.24; 6.15]
Q4 (wealthier)	146	79 (54.1)	0.60* [0.37; 0.97]	0.17* [0.03; 0.92]
Q5 (most wealthy)	141	87 (61.7)	0.82 [0.49; 1.34]	0.48 [0.09; 2.54]
Can read	701			
No	599	373 (62.3)	1	1
Yes	102	74 (72.5)	1.60* [1.01; 2.55]	10.27* [1.52; 69.28]
Age of pregnancy at the first ANC	690			
4 months or more	384	229 (59.6)	1	1
3 months or less	306	209 (68.3)	1.46* [1.06; 2.00]	1.58 [0.53; 4.72]
Knowledge of the usefulness of SP	700			
Incorrect answer	641	399 (62.2)	1	1
Prevention of malaria	59	47 (79.7)	2.38** [1.24; 4.57]	1.32 [0.2; 8.76]
Knowledge of the recommended SP dose	701			
Incorrect answer	475	250 (52.6)	1	-
Know the recommended dose	226	197 (87.2)	6.11*** [3.98; 9.39]	-
SP payment number	249			
1 time	84	16 (19.0)	1	1
2 times	70	66 (94.3)	70.13*** [22.28; 220.76]	139.18*** [32.94; 588.06]
3 times or more	95	92 (96.8)	130.33*** [36.52; 465.18]	244.73*** [54.28; 1103.5]
Rurality of the village of residence	701			
Rural	419	287 (68.5)	1	1
Less rural	282	160 (56.7)	0.60** [0.44; 0.82]	0.17** [0.05; 0.57]

^*^ = p < 0,05; ** = p < 0,01; *** = p ≤ 0,001

*OR* Odds Ratio, *Ora* Adjusted Odds Ratio

### Equity and distance to CHC

**Fig. 2:**
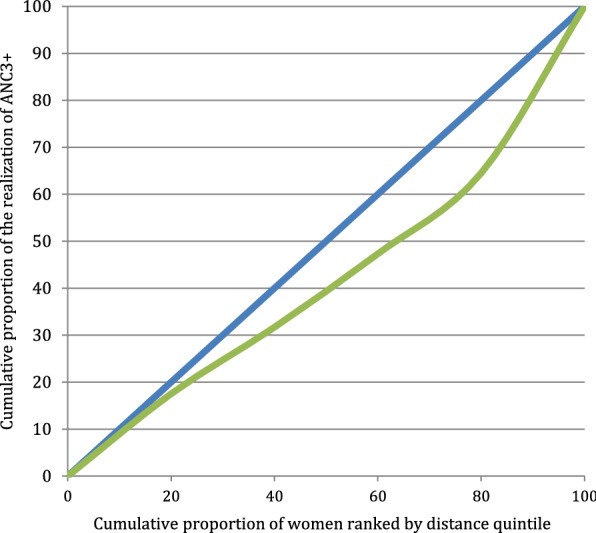
Curve of concentration of the realization of ANC3 + in the women surveyed in 2016, Sélingué, Mali

**Fig. 3:**
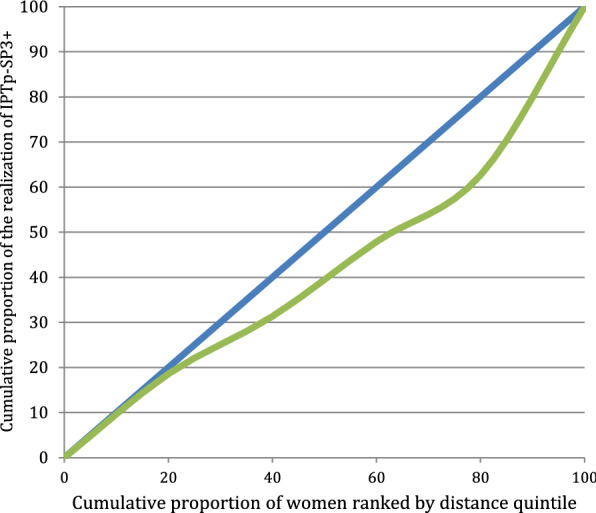
Curve of concentration of the realization of IPTp-SP3 + in the women surveyed in 2016, Sélingué, Mali

Figures 2 and 3 present the results of the equity analysis for completeness of adherence to ANC3 + and IPTp-SP3 + under the influence of distance from women's home villages to the CHC. Women close to the CHC were more likely to complete ANC3 + and IPTp-SP3 + than those far from the CHC, although this inequity was not statistically significant (Table 4).

**Table 4:** Concentration index of the realization of ANC3 + and IPTp-SP3 + in the women surveyed in 2016, Sélingué, Mali

Concentration index (CIndex)	Variance of CIndex	Standard error	t Statistic	95% Confidence interval (CI)
ANC3 +				
0.1641	0.0072	0.0850	1.9306	[− 0.0025; 0.3307]
IPTp-SP3 +				
0.1584	0.0096	0.0980	1.6168	[− 0.0336; 0.3504]

## Discussion

The same selection process of women as in the MIS and the DHS-V was used [10, 13]. ANC3 + as a dependent variable was chosen because from three ANC visits it is possible to have an adherence of IPTp-SP3 + [4–6]. This choice allowed women who had missed opportunities to benefit from IPTp-SP to be excluded from the determinants analysis, as Hurley reported [12].

### Frequency of ANC3 + and IPTp-SP3 + 

ANC is the entry point for the women included in this study to benefit from the WHO recommended intervention package [4, 6, 38] for their protection, and that of their foetus, as concluded the study by Hill et al*.* [16] and others [12, 47]. The frequency of ANC1 + (87.8%) in this study was high compared to the 78.5% ANC1 + found in the fifth DHS of Mali, in the Sikasso region [14], and the 78.3% found in the meta-analysis of van Eijk et al*.* [23]. It is also higher than the 81% in Hill et al*.* [16]. However, it is similar to the 89% of the 2016 statistical yearbook [11]. The frequency of ANC3 + (90.8%) in this study was high compared to those found in some studies [11, 14, 16, 23].

The study found an IPTp-SP3 + frequency of 63.7% among women who completed ANC3 + , which is similar to the frequency that Muhumuza et al*.* found in Uganda [29]. This frequency was greater than the 38.5% found by Odjidja et al*.* [48], but less than the 71% found by Ibrahim et al*.* [49], both in Ghana in 2017. Furthermore, it is more than the frequencies of the 2015 MIS of 19% in Sikasso [13], the 19.1% of the 2016 statistical yearbook [11], and the frequencies found in other studies [12, 16, 23, 25, 26, 29, 46, 50, 51]. For instance, Hill et al*.* found 28.6% for IPTp-SP2 + and 47.8% for IPTp-SP1 in Segou, Mali [16].

The high frequency of IPTp-SP is related to several factors. Sélingué District is an area that has benefited from the presence of a research unit of the National Institute of Research in Public Health and the intervention of Non-Governmental Organizations (NGOs), including Borne Fonden. The implementation of the project "Integrated control of malaria based on an environmental and community base in agro-ecosystems of West Africa" ​​in 2012 and 2013 is also an influencing factor [52]. During this project, an awareness raising campaign on ANC was conducted through local radio stations [52]. Additionally, the Village Malaria Committees and Farmers Field school served as a means for information, education, and action [52]. The presence of CHWs and Village Health Volunteers (VHV) have also helped to raise awareness. Notably, in 2014, the CHWs conducted 1,660 home visits involving 5,464 people (69.6% of whom were women), 1,369 discussion and sharing sessions, and 544 counseling sessions for 16,486 people (82% of whom were women) [53].

Since the implementation of the policy of three or more doses of SP started in Sélingué in 2014, it is possible that the women surveyed obtained the number of doses they mentioned [6]. Many women surveyed recognized the three SP tablets that the investigators showed them. Some of them used its local name (*sumaya fura kisè saaba*). This recognition was not always followed by the awareness of its usefulness. The investigators did not say the name in advance, to make sure the woman recognized the pills. Women's assertions based on the Kappa concordance test obtained by Hill et al*.* in Segou in 2014 were considered [16]. After the current study, the WHO guidance changed from “at least four ANC visit” to “eight visits” [4, 6]. This change could influence future studies on the number of IPTp-SP.

This study may have also benefited from the policy shift in IPTp-SP, with the possibility of taking the medication up to the time of delivery, starting at four months of pregnancy. This could be an explanation of the observed frequency. Moreover, this is the first study that covered the whole district of Sélingué.

### Determinants of ANC3 + 

The participants who reported knowledge of the usefulness of taking SP and had started taking SP earlier in their pregnancies were more likely to benefit from ANC3 + compared to those who did not know of SP’s usefulness or who did not take it early in pregnancy. This theory is reinforced by the education and literacy level. Others, including Webster et al*.,* and Faye et al*.,* reported education as a predictor of ANC compliance in their studies [17, 21].

Distance to facility was a barrier to the completeness of ANC3 + [12, 54, 55]. This is why women in villages with a CHC were significantly more likely to achieve their ANC3 + as compared to those in villages without a CHC. An analysis of data from 10 West African countries in 2016 found that distance, in addition to poverty, level of education, and rurality of the village of residence, were barriers to ANC utilization, as well as its effectiveness [56]. Additionally, the 2016 Statistical Yearbook identified the following factors to explain low rates of ANC: the late initiation of reproductive health services, inadequate completion of monitoring forms, insufficient qualified staff in peripheral reproductive health services, insufficient active research, inadequate reception, and poor communication [11].

### Determinants of the observance of IPTp-SP3 + 

The 12.2% of women who did not have any antenatal visits would not receive IPTp-SP. Among those who had received ANC, 13.5% had not received SP. ANC appears as a fundamental barrier to the completeness of IPTp-SP3 + . Furthermore, not receiving ANC was identified as a barrier to the completeness of IPTp-SP3 + in a study conducted by Hurley et al*.* in 2016 [12]. Similarly, a study by Sangaré et al*.* found that 68.7% of women eligible for IPTp-SP2 did not take any or simply had a single dose of IPTp-SP [19]. Yaya et al*.* found that women who completed ANC4 + had higher odds of taking IPTp-SP drugs (OR = 1.656, 95%CI = 1.194–2.299) [57]. Similar result was found by Mushi et al*.* [58].

The results show that relatively wealthy women were significantly (40%) less likely to complete IPTp-SP3 + compared to poor women, as opposed to what is generally reported in the literature [27], where adherence is proportional to wealth level. One explanation of this may be that more wealthy women attend private clinics for their ANC.

Women who were educated, who knew the usefulness of SP and the correct dose of SP were significantly more likely to complete IPTp-SP3 + . Similar findings were reported by Hill et al*.* in Mali, Ameh et al*.* and Onyeneho et al*.* in Nigeria, Kibusi et al*.* and Mushi et al*.* in Tanzania, and Yaya et al*.* in Ivory Coast [16, 25, 27, 57–59]. The compliance with IPTp-SP3 + , therefore, appears proportional to the level of education. In their systematic review and meta-analysis, Hill et al*.* found that having the correct information on IPTp-SP increased the likelihood of taking the SP [24]. In the same way, Pell et al*.* found that lack of knowledge was a barrier to IPTp-SP [60].

This study found that starting ANC early was a good indicator of IPTp-SP3 + compliance, which reinforces findings from other studies [16, 24, 27]. However, with the WHO recommendations [6], a woman can have three doses (IPTp-SP3 +) even if she started her ANC at 6–7 months, taking SP every month, provided there is regular attendance at ANC visits after this late start. In this case, this indicator could, in the future, no longer serve as an adequate indicator of compliance.

The DOT of SP, as recommended by WHO and PNLP [4–6], was not widely practiced in Sélingué, compared to findings of other studies [26, 61]. Half of the participants this study had practiced the DOT of SP. The other half took their SP home for self-administration, in absence of an observer, contrary to the recommendations [4–6]. The absence of observed treatment represents a barrier to IPTp-SP3 + compliance [16, 17, 24–26].

Few women were aware that SP can be taken on an empty stomach. Unavailability of drinking fountain or cups by the fountain, as well as asking women if they had eaten prior to coming to the health centre, influenced the non-observance of DOT of IPTp-SP. This suggests lack of knowledge of health workers on the guidelines for the administration of SP as identified by van Eijk et al*.* [23]. Similarly, Mubyazi et al. [62] found that the lack of clean water and cups were barriers to the DOT of SP. In another study, some pregnant women brought their own water bottle to the health centre to take SP, which enabled a barrier to DOT of SP [26]. Other authors found the positive influence of the availability of drinking water on the DOT of SP [17, 28], hence the availability of clean water and cups is recommended to improve the DOT [50]. Additionally, as noncompliance with DOT of IPTp-SP guidelines has been identified in several studies [12, 15, 16, 19, 24], a need for competence building is largely suggested [18, 24, 27, 63].

Distance was also identified as a barrier to IPTp-SP3 + , and was confirmed by the equity analysis. Women in CHC village sites were more likely to complete IPTp-SP3 + compared to women in villages far from the CHC. These findings are supported by those in the study by Hurley et al*.* [12]. However, the findings from this study conflict with those of Hill et al. which suggests that women in villages close to CHC were less likely to complete IPTp-SP [16].

Purchasing SP was a facilitator of IPTp-SP3 + completeness. This trend was confirmed by the number of payments mentioned by the participants. The higher the number of payments for SP, the more likely the woman was to complete IPTp-SP3 + , which appeared paradoxical in the local context. The national guidelines suggest that SP must be delivered to pregnant women free of charge [4, 6, 64]. However, paying for SP did not appear as a barrier. Payment of SP was reported by women in the study carried out by Klein et al. [15] in Koulikoro and Sikasso, Mali, which support the comments made by the participants in this study. Klein et al*.* found that payment was one barrier for the IPTp-SP2 + and that even staff were confused about IPTp-SP being free of charge [15]. Payment for SP linked to IPTp-SP completeness may be due to knowledge of its usefulness, which was observed to correlate positively with knowledge of the recommended doses, as the following: 1) the husbands or attendants were in charge of the payment of the prescription given to the women; 2) difficulties to differentiate between paid and free drugs; 3) the husbands or attendants did not share with women the information on the policy of offering SP free of cost; 4) lack of clear explanations of free medicines by staff, or 5) free drugs are not available from the ANC agent but rather from the pharmacy. Some of these explanations are corroborated by the studies of Hurley et al*.* and Hill et al*.* [12, 24]. It should be noted that the guidelines recommend both predisposing the free SP in the ANC units instead of the pharmacy and, on the other hand, making the separate SP prescription [64]. This is not always done at the CHC.

The wide confidence interval of SP payment number (Table 3) suggests that more exploration for this relationship is needed.

### Equity analysis

Findings for the equity analysis suggested that distance impacts the completeness of ANC3 + and IPTp-SP3 + . The curves (Figs. 2 and 3) indicated inequities in favour of women in the villages closest to the CHCs, especially after the first quintile of distance. Women nearer were more likely to complete ANC3 + and IPTp-SP3 + than those who were distant.

### Study limitations

The IPTp-SP questions did not specify exactly which dose was targeted because it was not standardized on a specific dose, such as the first dose taken or the last dose taken. The woman could answer on any dose. The IPTp-SP questions were asked only to women who said they had received ANC. Those who did not attend ANC could have interesting information, especially multiparas, hence a potential source of selection bias. The cross-sectional nature of the study and the subjective responses of the women surveyed could introduce information biases, not only by the women themselves, but also by the interviewers due to the length of the questionnaire, the time needed for the interview, and some skips or connection problems in the Magpi software used for electronic data collection. Lastly, the availability of SP stock in the health centres was not evaluated. However, few women mentioned SP stock shortage at the CHC as the reason for not obtaining and taking SP.

## Conclusion

Despite the high frequency of IPTp-SP3 + in this study, Mali is still far from the goal of universal coverage of pregnant women. Several critical factors in achieving this goal were identified. The positive factors include the level of education, the ability to read, the early start of ANC, the knowledge of the usefulness of the drug, the recommended dose of the drug and the observance of DOT at the CHC. Some of the barriers include the distance to the health facility, the lack of implementation of ANC, and the unavailability of water for DOT. This study shows that a successful implementation of the IPTp strategy can be achieved by improving access to prenatal care at community health facilities, and strengthening patient-provider communication, to ensure adequate knowledge on dosing and IPTp-SP benefits to mother and child.

## Data Availability

The datasets used and/or analysed during the current study are available from the corresponding author on reasonable request.

## Abbreviations

ANC: Antenatal care or antenatal visits
CHC: Community Health Centre
CHW: Community Health Workers
CI: Confident Interval
CIndex: Concentration index
CR: Community relays / volunteers
DHS: Demographic and Health Surveys (DHS)
DOT: Direct Observed Therapy
IPTp-SP: Intermittent Preventive Treatment with Sulfadoxine-pyrimethamine
ITN: Insecticide-Treated Nets
MIS: Malaria Indicators Survey
NGOs: Non-Governmental Organizations
OR: Odds Ratio
ORa: Adjusted Odds Ratio
PNLP: National Malaria Control Programme
SPSS: Statistical Package for the Social Sciences
VHV: Village Health Volunteers
WHO: World Health Organization
